# A neutralizing human antibody binds to the N-terminal domain of the Spike protein of SARS-CoV-2

**DOI:** 10.1126/science.abc6952

**Published:** 2020-06-22

**Authors:** Xiangyang Chi, Renhong Yan, Jun Zhang, Guanying Zhang, Yuanyuan Zhang, Meng Hao, Zhe Zhang, Pengfei Fan, Yunzhu Dong, Yilong Yang, Zhengshan Chen, Yingying Guo, Jinlong Zhang, Yaning Li, Xiaohong Song, Yi Chen, Lu Xia, Ling Fu, Lihua Hou, Junjie Xu, Changming Yu, Jianmin Li, Qiang Zhou, Wei Chen

**Affiliations:** 1 Beijing Institute of Biotechnology, Academy of Military Medical Sciences (AMMS), Beijing 100071, China.; 2 Key Laboratory of Structural Biology of Zhejiang Province, Institute of Biology, Westlake Institute for Advanced Study, School of Life Sciences, Westlake University, Hangzhou 310024, Zhejiang Province, China.; 3 Beijing Advanced Innovation Center for Structural Biology, Tsinghua-Peking Joint Center for Life Sciences, School of Life Sciences, Tsinghua University, Beijing 100084, China.

## Abstract

Developing therapeutics against severe acute respiratory syndrome coronavirus 2 (SARS-CoV-2) could be guided by the distribution of epitopes, not only on the receptor binding domain (RBD) of the Spike (S) protein but also across the full Spike (S) protein. We isolated and characterized monoclonal antibodies (mAbs) from 10 convalescent COVID-19 patients. Three mAbs showed neutralizing activities against authentic SARS-CoV-2. One mAb, named 4A8, exhibits high neutralization potency against both authentic and pseudotyped SARS-CoV-2 but does not bind the RBD. We defined the epitope of 4A8 as the N-terminal domain (NTD) of the S protein by determining with cryo–eletron microscopy its structure in complex with the S protein to an overall resolution of 3.1 angstroms and local resolution of 3.3 angstroms for the 4A8-NTD interface. This points to the NTD as a promising target for therapeutic mAbs against COVID-19.

## Hitting SARS-CoV-2 in a new spot

A key target for therapeutic antibodies against severe acute respiratory syndrome coronavirus 2 (SARS-CoV-2) is the spike protein, a trimeric protein complex with each monomer comprising an S1 and an S2 domain that mediate binding to host cells and membrane fusion, respectively. In addition to the receptor binding domain (RBD), S1 has an N-terminal domain (NTD). In searching for neutralizing antibodies, there has been a focus on the RBD. Chi *et al.* isolated antibodies from 10 convalescent patients and identified an antibody that potently neutralizes the virus but does not bind the RBD. Cryo–electron microscopy revealed the epitope as the NTD. This NTD-targeting antibody may be useful to combine with RBD-targeting antibodies in therapeutic cocktails.

*Science*, this issue p. 650

## Teaser

A region outside of the receptor binding domain of SARS-CoV-2 is targeted by a neutralizing antibody.

The global outbreak of COVID-19 has emerged as a severe threat to human health (*1*–*3*). COVID-19 is caused by a novel coronavirus, the severe acute respiratory syndrome coronavirus 2 (SARS-CoV-2), which is an enveloped, positive-strand RNA virus that causes symptoms such as cough, headache, dyspnea, myalgia, fever, and severe pneumonia in humans (*1*, *3*–*5*).

SARS-CoV-2 is a member of the β coronavirus genus, which also contains SARS-CoV and MERS-CoV, which caused epidemics in 2002 and 2012, respectively (*6*, *7*). SARS-CoV-2 shares about 80% sequence identity to SARS-CoV and uses the same cellular receptor, angiotensin-converting enzyme 2 (ACE2) (*8*–*16*).

The trimeric S protein decorates the surface of coronavirus and plays a pivotal role during viral entry (*17*, *18*). During infection, the S protein is cleaved into the N-terminal S1 subunit and C-terminal S2 subunit by host proteases such as TMPRSS2 (*18*, *19*) and changes conformation from the prefusion to the postfusion state (*20*). S1 and S2 comprise the extracellular domain (ECD; 1 to 1208 amino acids) and a single transmembrane helix and mediate receptor binding and membrane fusion, respectively (*16*). S1, which consists of the N-terminal domain (NTD) and the receptor binding domain (RBD), is critical in determining tissue tropism and host ranges (*21*, *22*). The RBD is responsible for binding to ACE2, whereas the function of NTD is not well understood. In some coronaviruses, the NTD may recognize specific sugar moieties upon initial attachment and might play an important role in the prefusion-to-postfusion transition of the S protein (*23*–*26*). The NTD of the MERS-CoV S protein can serve as a critical epitope for neutralizing antibodies (*26*).

The SARS-CoV-2 S protein–targeting monoclonal antibodies (mAbs) with potent neutralizing activity are a focus in the development of therapeutic interventions for COVID-19 (*27*–*29*). Many studies reported the functions and structures of SARS-CoV-2–neutralizing antibodies that target the RBD and inhibit the association between the S protein and ACE2 (*28*–*34*). The RBD-targeting antibodies, applied individually, might induce resistance mutations in the virus (*26*). Antibodies that target non-RBD epitopes might be added to antibody cocktail therapeutics for SARS-CoV-2. We thus sought to identify antibodies to different regions of the S protein and to the Nucleocapsid (N) protein.

## Results

### Isolation of human mAbs from memory B cells and plasma B cells

**Fig. 1:**
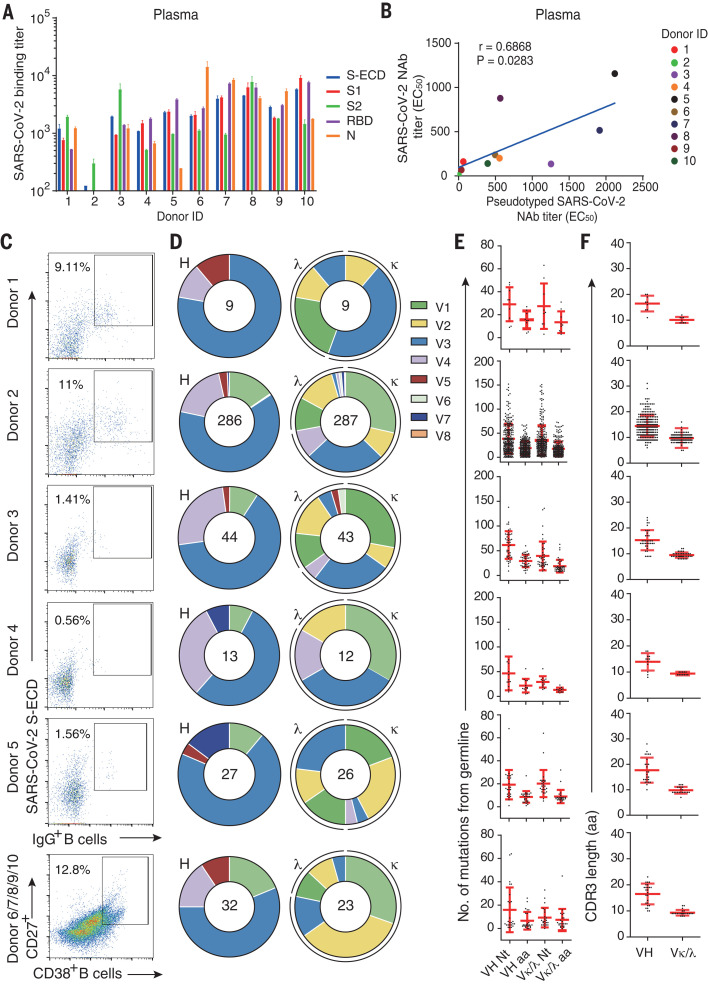
Isolation of antigen-specific mAbs from convalescent patients of SARS-CoV-2. (**A**) Reactions of plasma to SARS-CoV-2 proteins. S-ECD (extracellular domain of S protein), S1, S2, RBD (receptor binding domain), and N protein were used in ELISA to test the binding of plasma. Plasma of heathy donors were used as control, and cut-off values were calculated as optical density (OD) 450 of control × 2.1. Data were shown with mean and SD of a representative experiment. (**B**) The correlations between the authentic SARS-CoV-2 neutralizing antibody (NAb) titers and the pseudotyped SARS-CoV-2 NAb titers in plasma. Neutralizing assays of plasma against authentic SARS-CoV-2 were performed by using Vero E6 cells, and neutralization against pseudotyped SARS-CoV-2 were determined by using ACE2-293T cells. The correlations were calculated by means of Pearson correlation test in Graphpad 7.0. (**C**) Flow cytometry sorting from PBMCs of 10 convalescent patients. (**D**) Distribution of V gene families in heavy and light chains of all distinct clones (the total number is shown in the center of the pie charts) for each donor. (**E**) The number of amino acid (AA) and total nucleotide (Nt) mutations from the germline of all clonal sequences identified in (D) is shown. (**F**) CDR3 amino acid lengths of VH and VL of all clonal sequences identified in (D).

To isolate mAbs and analyze the humoral antibody responses to SARS-CoV-2, we collected plasma and peripheral blood mononuclear cells (PBMCs) from 10 Chinese patients who had recovered from SARS-CoV-2 infection. The age of donors ranges from 25 to 53 years. The interval from disease confirmation date to blood collection date ranged from 23 to 29 days for patients 1 to 5 and 10 to 15 days for patients 6 to 10 (table S1). We evaluated the titers of binding antibodies in plasma to different fragments of the SARS-CoV-2 S protein—including the full ECD, S1, S2, and the RBD—and to the N protein. Plasma from all the patients except donor 2 bound to all five SARS-CoV-2 protein segments, whereas that from donor 2 recognized S-ECD and S2 only (Fig. 1A). The neutralizing capacities of plasma against authentic SARS-CoV-2 and HIV-vectored pseudotyped SARS-CoV-2 are correlated [correlation coefficient (*r*) = 0.6868, *P* < 0.05] (Fig. 1B). These results indicate that humoral immune responses were specifically elicited for all 10 patients during their natural infection with SARS-CoV-2.

To isolate S protein–specific mAbs, we first sorted the immunoglobulin G–positive (IgG^+^) memory B cells from PBMCs of convalescent patients 1 to 5 with flow cytometry, using S-ECD as the probe (Fig. 1C). The percentage of S-ECD–reactive IgG^+^ B cells ranges from 0.56 to 11%, as revealed with fluorescence activating cell sorting (FACS). To avoid losing B cells with low copies of S-ECD–specific receptors on cell surfaces, we sorted plasma B cells from mixed PBMCs derived from another five convalescent patients (patients 6 to 10) without using S-ECD protein as the probe in flow cytometry. The percentage of plasma B cells in CD3-CD19^+^ B cells was 12.8%, which is higher than the percentage of memory B cells in CD3-CD19^+^ B cells (Fig. 1C).

From the sorted B cells, we identified 9, 286, 43, 12, and 26 clones of single B cell from patients 1 to 5, respectively, and 23 clones of single B cell from the mixed PBMCs of patients 6 to 10 (Fig. 1D). The distribution of the sequenced heavy (IgH) gene families was comparable among the 10 donors, with *VH3* being the most commonly used VH gene, whereas different donors displayed variable preferences for the light chain (IgL) gene families (Fig. 1D). The combination of V3 and J4, V3 and D3, and D3 and J4 were the most common usage for the IgH gene family (fig. S1). The average mutations of amino acids per mAb from memory B cells ranged from 17.50 to 48.04 for donors 1 to 5, respectively, whereas mAbs from plasma B cells possessed an average of 13.99 amino acid mutations for donors 6 to 10 (Fig. 1E). Human antibodies elicited through repeated exposures to different antigens confer an average of 26.46 amino acid mutations per Ab, as previously reported (*35*). These results indicate that natural SARS-CoV-2 infection elicited high levels of somatic hypermutation (SHM) in memory B cells. The lengths of complementarity-determining region 3 (CDR3) for antibodies were similar among the donors, with average lengths of these CDR3 ranging from 13.9 to 17.7 for VH and 9.3 to 10.1 for VL (Fig. 1F). The CDR3 lengths of these mAbs were longer than that in antigen-specific immune receptors (means of 12.7 for VH and 6.5 for VL, respectively) reported previously (*36*).

### Binding profiles of SARS-CoV-2 S protein–specific human mAbs

**Fig. 2:**
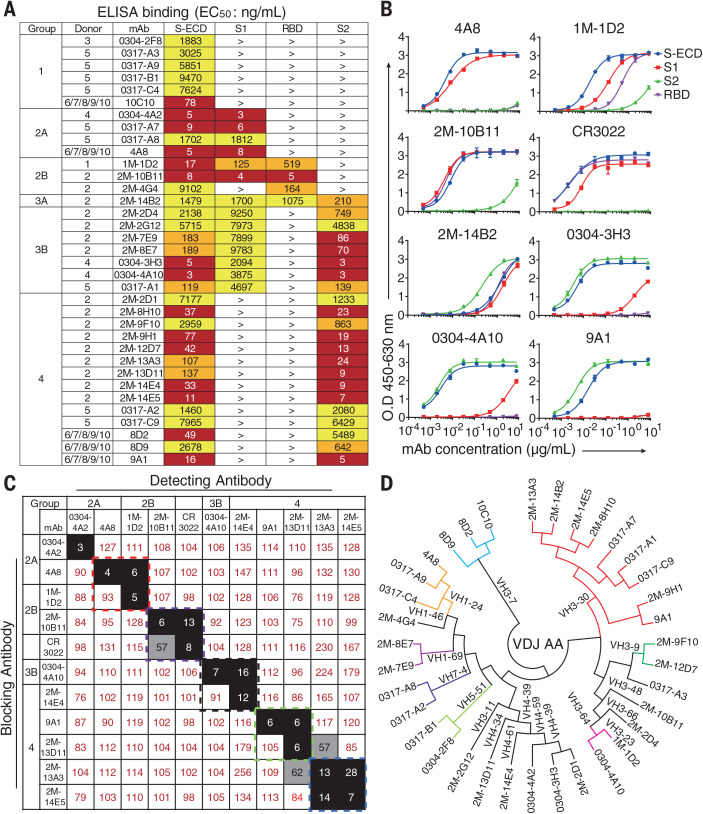
Binding profiles of Spike protein–specific mAbs. (**A**) Heatmap showing the binding of mAbs to different types of spike proteins determined by using ELISA. The EC_50_ value for each S-mAb combination is shown, with dark red, orange, yellow, or white shading indicating high, intermediate, low, or no detectable binding, respectively. EC_50_ values greater than 10,000 ng/ml are indicated (>). (**B**) Binding curves of representative mAbs. CR3022 is a control that was reported to bind SARS-CoV and SARS-CoV-2 RBD. Data were shown with mean and SD of a representative experiment. (**C**) Heatmap showing the competing binding of some representative S-reactive mAbs assayed in ELISA. Numbers in the box indicate the percentage binding of detecting mAb in the presence of the blocking antibody compared with the binding of detecting mAb in the absence of the blocking antibody. The mAbs were considered competing if the inhibiting percentage is <30% (black boxes with white numbers). The mAbs were judged to noncompete for the same site if the percentage is >70% (white boxes with red numbers). Gray boxes with black numbers indicate an intermediate phenotype (30 to ~70%). (**D**) Phylogenetic trees of all the S-specific mAbs.

To screen for S protein–specific antibodies, we determined the binding specificity using enzyme-linked immunosorbent assay (ELISA) for the 399 human mAbs sorted above. From donors 1 to 5, respectively, 1, 16, 1, 3, and 9 S-ECD–specific mAbs were identified. A total of 35 S-ECD–specific mAbs were identified from donors 6 to 10 (Fig. 2A). We further characterized domain specificities of the 35 mAbs with different fragments of the S protein, including S1, S2, and RBD (Fig. 2A). The S-reactive mAbs are classified into four major groups on the basis of their medium effective concentration (EC_50_) values (Fig. 2A). Group 1 recognizes only S-ECD. Group 2 recognizes S-ECD and S1, with subgroup 2A binding S-ECD and S1 and subgroup 2B binding S-ECD, S1, and RBD. Group 3 interacts with both S1 and S2, where subgroup 3A targets the RBD and subgroup 3B fails to bind the RBD. Group 4 recognizes S-ECD and S2. Only four mAbs recognize the RBD among the 35 S-specific mAbs (Fig. 2, A and B).

We performed a competition-binding assay using ELISA for several representative mAbs to determine whether there are overlapping antigenic sites between different mAbs, with CR3022 being used as a positive control mAb that reported to bind the SARS-CoV-2 RBD (Fig. 2C) (*37*). Among these mAbs, 4A8 in group 2A competed with 1M-1D2 in group 2B. Another RBD-reactive mAb, 2M-10B11 in group 2B, competed with CR3022, suggesting overlapped epitopes on RBD for these two mAbs. These results indicate that antibody responses elicited by natural SARS-CoV-2 infection were diverse in epitope recognition of S proteins.

To characterize the diversity in gene usage and affinity maturation, the phylogenetic trees of these S-ECD–specific mAbs were analyzed on the basis of the amino acid sequences of VHDJH and VLJL by using a neighbor-joining method in MEGA7 Software (*38*). Results indicate that the VH gene usage is very diverse among the 35 mAbs from 10 donors, with *VH3-30* being the most frequently used germline gene. There was no particularly favored VH gene identified among S1, S2, or RBD-reactive mAbs (Fig. 2D). The percentages of heavy chain variable gene sequence identity ranged from 40.9 to 97.6% in the 35 S-ECD–specific mAbs (fig. S2 and table S2).

### Neutralizing activities of SARS-CoV-2 S–specific human mAbs

**Fig. 3:**
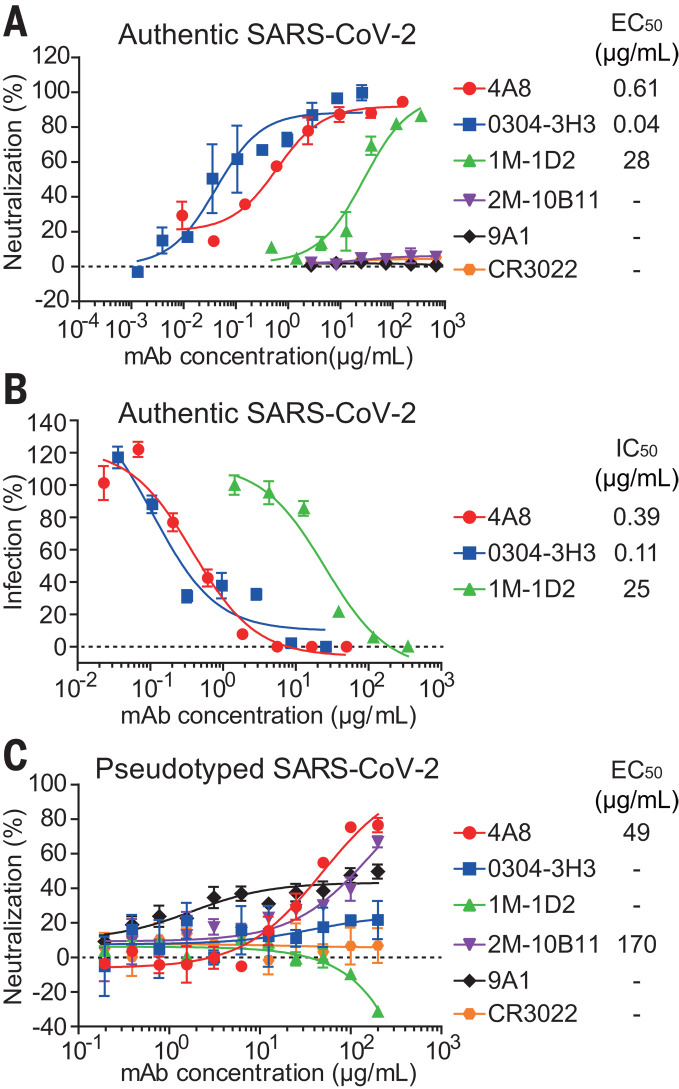
Neutralizing capacities of S-reactive mAbs. (**A**) Neutralization of S-reactive mAbs to authentic SARS-CoV-2 in Vero-E6 cells. (**B**) The authentic SARS-CoV-2 virus RNA load was determined in Vero-E6 cells treated with S-reactive mAbs by using quantitative PCR. Percent infection was calculated as the ratio of RNA load in mAb-treated wells to that in wells containing virus only. (**C**) Neutralization of S-reactive mAbs against HIV-vectored pseudotyped SARS-CoV-2 in ACE2-293T cells. Data were shown as mean ± SD of a representative experiment.

We first performed in vitro neutralization studies of the 35 S-ECD–specific mAbs using authentic SARS-CoV-2 in Vero-E6 cells (Fig. 3A). Of the 35 S-ECD–specific mAbs, only three mAbs neutralized authentic SARS-CoV-2. MAb 1M-1D2, 4A8, and 0304-3H3 exhibited medium to high neutralizing capacity with EC_50_ of 28, 0.61, and 0.04 μg/ml, respectively. As expected, the RBD-targeting control mAb, CR3022, failed to neutralize authentic SARS-CoV-2 (*37*). Moreover, although the CR3022-competing mAb, 2M-10B11, bound to the SARS-CoV-2 RBD with an EC_50_ of 5 ng/ml (Fig. 2A), it also failed to neutralize authentic SARS-CoV-2. These results suggest that binding affinities of mAbs against RBD do not correlate fully with the neutralizing abilities of mAbs. To further investigate the inhibitory activity of the three authentic SARS-CoV-2–neutralizing mAbs—4A8, 0304-3H3, and 1M-1D2—we tested the RNA load of authentic SARS-CoV-2 in Vero-E6 cells treated with each mAb using real-time quantitative polymerase chain reaction (PCR) (Fig. 3B). Consistent with the cytopathic effect (CPE) assay results (Fig. 3A), mAbs 0304-3H3 and 4A8 displayed higher inhibitory capacities than did 1M-1D2 (Fig. 3B).

We next performed luciferase reporter gene assays for all 35 S-binding mAbs using HIV-vectored pseudotyped SARS-CoV-2 (*39*), among which three mAbs exhibited neutralizing activity against the pseudotyped virus (Fig. 3C). 4A8 protected ACE2-293T cells with an EC_50_ of 49 μg/ml. Although mAb 2M-10B11 and 9A1 did not neutralize authentic SARS-CoV-2, 2M-10B11 protected against pseudotyped virus with an EC_50_ of 170 μg/ml, and 9A1 provided weak protection. To our surprise, neutralization by 0304-3H3 and 1M-1D2 was not observed (Fig. 3C). The inconsistency between the results for pseudotyped SARS-CoV-2 compared with authentic SARS-CoV-2 were also observed for mAbs against MERS-CoV (*40*, *41*) and may be caused by the different presentation of S protein resulted from the different environmental factors the viruses underwent, such as the cells used for the neutralizing assays or for the production of the pseudotyped or authentic virions (*42*). On the basis of these results, 4A8 is a potential candidate for the treatment of SARS-CoV-2 because it displayed strong neutralizing capacities against both authentic and pseudotyped SARS-CoV-2.

### Binding characterization of candidate mAbs

**Fig. 4:**
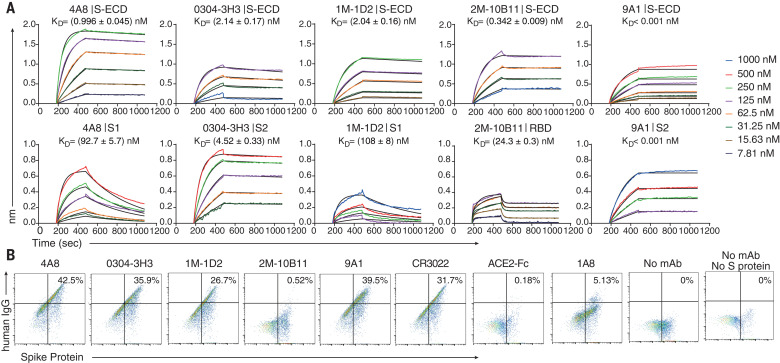
4A8 did not block the binding of Spike protein to ACE2 receptor. (**A**) BLI sensorgrams and kinetics of mAbs binding to S proteins. Global fitting curves are shown as black lines. The *K*_d_ were calculated by using a 1:1 binding model in Data Analysis Software 9.0, except for 2M-10B11, which used a heterogeneous ligand model owing to avidity effect. (**B**) The binding of S protein to human ACE2-overexpressing 293T cells were determined by means of flow cytometry. After the preincubation of S protein with each indicated mAb, the mAb-S mixtures were added to the ACE2-expressing cells. Cells were stained with anti-human IgG fluorescein isothiocyanate (mAb binding, *x* axis) and anti-His (S binding, *y* axis). Percentages of double-positive cells are shown. Control mAb CR3022 and 1A8 were previously reported to bind SARS-CoV RBD and Marburg glycoprotein, respectively, and ACE2-Fc protein was a human ACE2 protein conjugated with human Fc.

To determine the possible neutralizing mechanism of the mAbs, we determined the binding affinities of the five mAbs with potential neutralizing activity against different segments of the S protein—including the full S-ECD and domains S1, S2, and RBD—using biolayer interferometry (BLI). All five tested mAbs bound to S-ECD with high affinity; equilibrium dissociation constants (*K*_d_) were less than 2.14 nM (Fig. 4A). 4A8 and 1M-1D2 bound to S1 with *K*_d_ of 92.7 and 108 nM, respectively, whereas 0304-3H3 and 9A1 targeted S2 with *K*_d_ of 4.52 and <0.001 nM, respectively (Fig. 4A, bottom). Moreover, 2M-10B11 bound the RBD with *K*_d_ of 24.3 nM, which was obtained by using heterogeneous ligand model owing to the avidity effects (Fig. 4A, bottom).

To investigate whether these mAbs block the binding of S protein to ACE2, we performed flow cytometry using human embryonic kidney (HEK) 293T cells expressing human ACE2. As expected, only 2M-10B11 among the five mAbs and ACE2-Fc prevented S protein from binding to ACE2. In the presence of 2M-10B11, only 0.52% of cells were double positive for IgG and S protein (Fig. 4B). CR3022, which competes with 2M-10B11, did not block the binding of S to ACE2. The control mAb 1A8, targeting the Marburg glycoprotein, did not interfere with the binding either, and the 5.13% of double positives may be due to the nonspecific binding of 1A8 to S protein. 4A8 also failed to interfere with the binding of the S protein to ACE2.

### Cryo-EM structure of the complex between 4A8 and S-ECD

The mAb 4A8 was overexpressed and purified by Protein A resin, and the S-ECD of SARS-CoV-2 was purified through M2 affinity resin and size exclusion chromatography (SEC). 4A8 and S-ECD protein were mixed and incubated at a stoichiometric ratio of ~1.2 to 1 for 1 hour and applied to SEC to remove excess proteins (fig. S3A). The fraction containing the complex was concentrated for cryo–electron microscopy (cryo-EM) sample preparation.

**Fig. 5:**
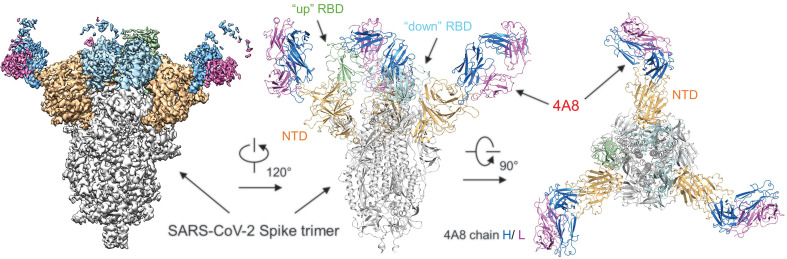
Cryo-EM structure of the 4A8 and S-ECD complex. The domain-colored cryo-EM map of the complex is shown on the left, and two perpendicular views of the overall structure are shown on the right. The heavy and light chains of 4A8 are colored blue and magenta, respectively. The NTDs of the trimeric S protein are colored orange. The one “up” RBD and two “down” RBDs of trimeric S protein are colored green and cyan, respectively.

To investigate the interactions between 4A8 and the S protein, we solved the cryo-EM structure of the complex at an overall resolution of 3.1 Å (Fig. 5 and movie S1). Details of cryo-EM sample preparation, data collection and processing, and model building can be found in in the supplementary materials, materials and methods (figs. S3 to S5). The S protein exhibits asymmetric conformations similar to the previously reported structures (*21*, *22*), with one of three RBDs in “up” conformation and the other two RBDs in “down” conformation (Fig. 5).

### Recognition of the NTD by 4A8

In the S protein–4A8 complex, each trimeric S protein is bound with three solved 4A8 Fabs, each of which interacts with one NTD of the S protein. Despite the different conformations of the three S protein protomers, the interface between 4A8 and each NTD is identical (Fig. 5 and fig. S3I). The map quality at the NTD-4A8 region was improved through focused refinement to a local resolution of 3.3 Å, enabling reliable analysis of the interactions between the NTD and 4A8.

Association with 4A8 appears to stabilize the NTD epitope, which is invisible in the reported S protein structure alone (*21*, *22*). Supported by the high resolution of NTD, we were able to build the structural model for five new loops for NTD, designated N1 (residues 14 to 26), N2 (residues 67 to 79), N3 (residues 141 to 156), N4 (residues 177 to 186), and N5 (residues 246 to 260), among which the N3 and N5 loops mediate the interaction with 4A8 (fig. S5A). Besides, three new glycosylation sites (Asn^17^, Asn^61^, and Asn^149^) on the NTD are identified in this structure (fig. S6).

**Fig. 6:**
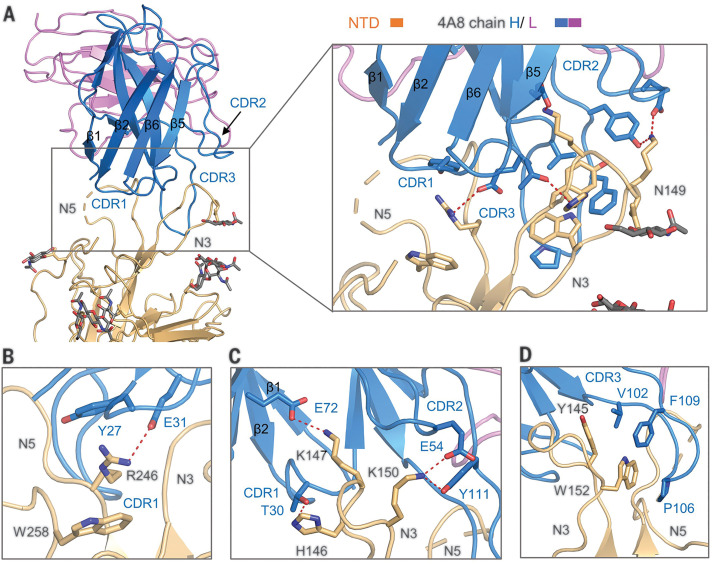
Interactions between the NTD and 4A8. (**A**) Extensive hydrophilic interactions on the interface between NTD and 4A8. Only one NTD-4A8 is shown. (**B** to **D**) Detailed analysis of the interface between NTD and 4A8. Polar interactions are indicated by red dashed lines. The residues involved in hydrophobic interactions are presented as spheres.

The heavy chain of 4A8 mainly participates in binding to the NTD mainly through three complementarity-determining regions (CDRs), named CDR1 (residues 25 to 32), CDR2 (residues 51 to 58), and CDR3 (residues 100 to 116) (Fig. 6A and fig. S5B). The interface is constituted by an extensive hydrophilic interaction network, and the buried surface area at the 4A8-NTD interface is 832 Å^2^. Arg^246^ on the N5 loop of the NTD represents one docking site, which is stabilized by Trp^258^, simultaneously interacting with Tyr^27^ and Glu^31^ of 4A8 on CDR1 (Fig. 6B). On the N3 loop of the NTD, Lys^150^ and Lys^147^ respectively form salt bridges with Glu^54^ and Glu^72^ of 4A8 (Fig. 6C). Lys^150^ is also hydrogen (H)–bonded with 4A8-Tyr^111^, while His^146^ forms a H-bond with 4A8-Thr^30^ (Fig. 6C). In addition to the hydrophilic interactions, Trp^152^ and Tyr^145^ on the N3 loop of the NTD also interact with Val^102^, Pro^106^, and Phe^109^ on the CDR3 of 4A8 through hydrophobic and/or π-π interactions (Fig. 6D). Additionally, the glycosylation site of Asn^149^ on the NTD is close to the 4A8-NTD interface, of which N-glycans might participate in the interactions on the interface (Fig. 6A and fig. S6).

## Discussion

There is an urgent need for prophylactic and therapeutic interventions for SARS-CoV-2 infections given the ongoing COVID-19 pandemic. Our work reveals that naturally occurring human SARS-CoV-2 mAbs isolated from the B cells of 10 recovered donors are diverse in gene usage and epitope recognition of S protein. The majority of the isolated mAbs did not recognize the RBD, and all the mAbs that neutralize authentic SARS-CoV-2 failed to inhibit the binding of S protein to ACE2. These unexpected results suggest the presence of other important mechanisms for SARS-CoV-2 neutralization in addition to suppressing the viral interaction with the receptor.

The S1-targeting mAb 4A8 does not block the interaction between ACE2 and S protein but exhibits high levels of neutralization against both authentic and pseudotyped SARS-CoV-2 in vitro. Many neutralizing antibodies against SARS-CoV-2 were reported to target the RBD of the S protein and block the binding between RBD and ACE2 (*28*–*30*, *32*–*34*). Our results show that 4A8 binds to the NTD of S protein with potent neutralizing activity. Previous study has shown that mAb 7D10 could bind to the NTD of S protein of MERS-CoV probably by inhibiting the RBD-DPP4 binding and the prefusion-to-postfusion conformational change of S protein (*26*). We aligned the crystal structure of 7D10 in complex with the NTD of S protein of MERS-CoV with our complex structure and found that the interfaces between the mAb and the NTDs are partially overlapped (fig. S7). 7D10 may inhibit the interaction between MERS-CoV and DPP4 through its light chain, which is close to the RBD. In our complex, the light chain of 4A8 is away from the RBD (fig. S7). Therefore, we speculate that 4A8 may neutralize SARS-CoV-2 by restraining the conformational changes of the S protein. Furthermore, sequence alignment of the S proteins from SARS-CoV-2, SARS-CoV, and MERS-CoV revealed varied NTD surface sequences that are respectively recognized by different mAbs (fig. S8).

This work reports a fully human neutralizing mAb recognizing a vulnerable epitope of NTD on S protein of SARS-CoV-2, functioning with a mechanism that is independent of receptor binding inhibition. Combination of 4A8 with RBD-targeting antibodies may avoid the escaping mutations of the virus and serve as promising “cocktail” therapeutics. The information obtained from these studies can be used for development of the structure-based vaccine design against SARS-CoV-2.

## Supplementary Materials

science.sciencemag.org/content/369/6504/650/suppl/DC1 Materials and Methods Figs. S1 to S7 Tables S1 to S4 References (*43*–*66*) Movie S1 MDAR Reproducibility Checklist
